# Bilateral Lateral Rectus Recession in Duane Retraction Syndrome Type II: A Case Report

**DOI:** 10.7759/cureus.35351

**Published:** 2023-02-23

**Authors:** Saif Alobaisi, Azam I Alromaih

**Affiliations:** 1 Pediatric Ophthalmology Department, King Abdullah Specialist Children Hospital, Riyadh, SAU; 2 College of Medicine, King Abdulaziz Medical City, Riyadh, SAU

**Keywords:** surgery, exotropia, strabismus, lateral rectus recession, duane retraction syndrome

## Abstract

A two year presented to the clinic with abnormal head posture and right-sided face turn since birth. On examination, he showed a large right face turn of 40° while concentrating on a near target. His ocular motility assessment showed a -4 limitation of adduction in the left eye with 40 prism diopters (PD) exotropia and grade 1 globe retraction of the left eye. He was diagnosed with type II Duane retraction syndrome (DRS) in the left eye and planned for lateral rectus recession of both eyes. Postoperatively, the patient was orthotropic at distance and near in primary gaze with resolved face turn and improvement of limitation of adduction to -2, but some limitation of abduction -1 in the left eye was observed. Herein, we discuss the clinical features, etiologies, tailored evaluation, and management for type II DRS patient.

## Introduction

Duane retraction syndrome (DRS) is a rare congenital ocular motility disorder characterized by frequent limitation of abduction with a narrowing of the palpebral fissure, retraction of the globe, and upshoot or downshoot on attempts of adduction [1]. The prevalence of DRS among strabismus patients is 5% and usually affects females more than males [1]. Also, DRS is commonly unilateral and affects the left eye more than the right eye [1]. Most DRS cases (90%) are sporadic, with the remaining 10% being familial [2]. Most researchers agree that the characteristic findings are best explained by a paradoxical innervation of the lateral rectus muscle, resulting in a co-contraction of the horizontal rectus muscles [2]. However, many theories regarding the etiology and pathogenesis of DRS have been proposed, including agenesis of the abducens nucleus [2]. DRS can be classified, according to Huber, into three types [1]. Type I DRS is the most common, accounting for 70-80% of all cases, followed by type III DRS, which accounts for 15%, and the least common is type II DRS, which accounts for 7% [1]. Type II DRS is characterized by limited adduction, primary position exotropia of the affected eye, and normal or slightly limited abduction [1]. The diagnosis of type II DRS is primarily clinical [1]. The management of DRS type II usually involves lateral rectus recession [3]. The primary indication for surgery is the correction of primary position deviation and abnormal face turn [3].

## Case presentation

**Figure 1:**
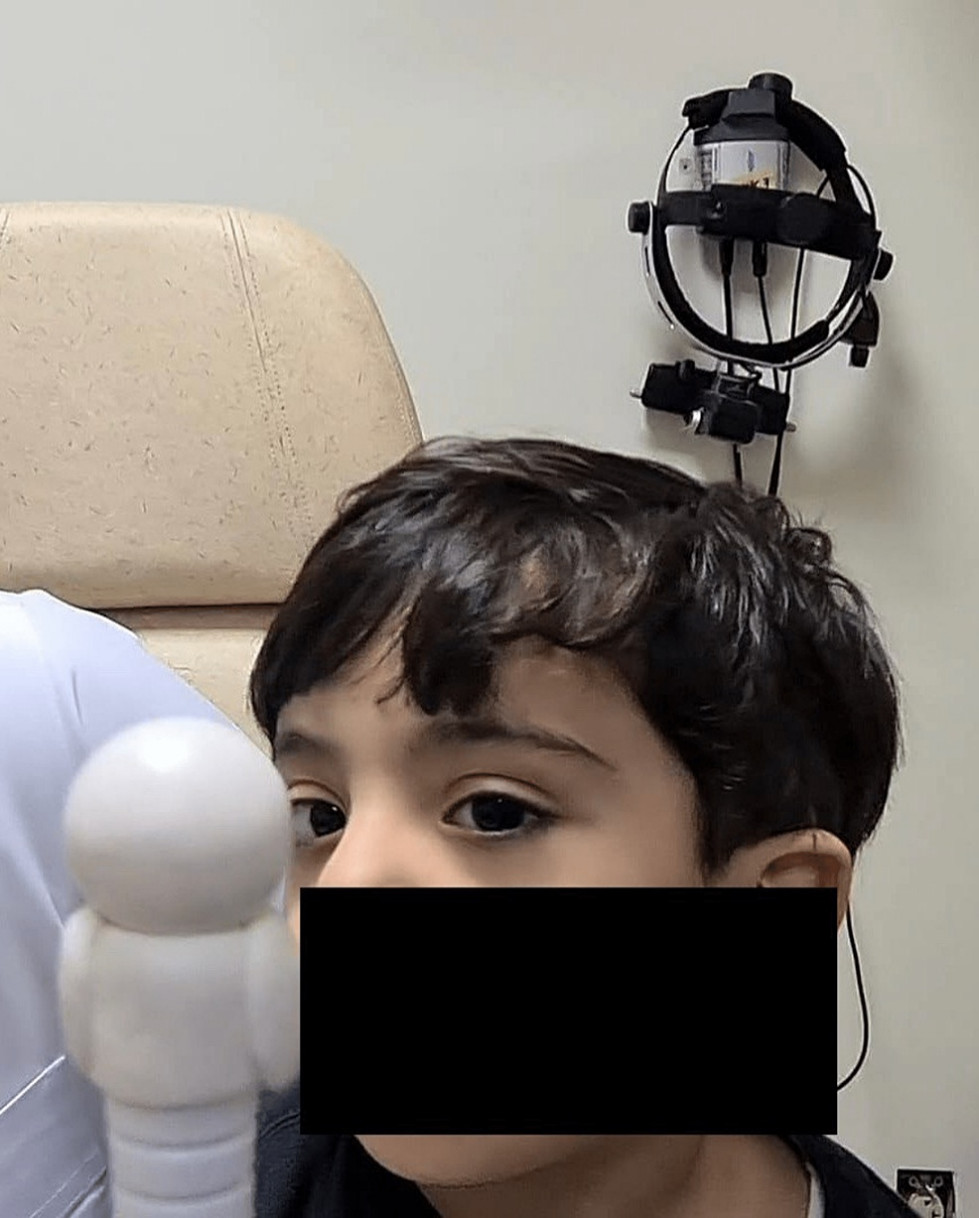
Preoperative assessment showing abnormal head posture with right large face turn

**Figure 2:**
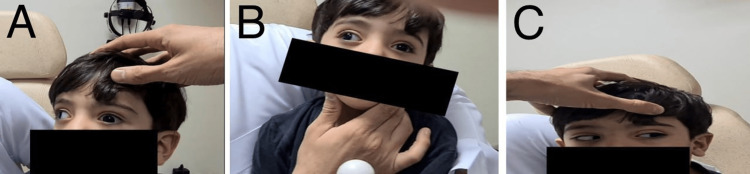
Preoperative assessment A. Limitation of adduction B. Large angle of exotropia in primary position C. Orthotropia in left gaze

A two-year-old boy was presented to our hospital due to a large face turn to the right side since birth. He has no history of ocular trauma or ocular surgery, and his birth and development were normal. The ophthalmologic evaluation showed a large right-sided face turn of 40° while concentrating at a near target (Figure 1). His vision assessment showed a clear preference toward the right eye, although he still maintains a large face turn with an abnormal head posture. Cycloplegic refraction showed +2.5 diopter sphere (DS) and -2.00 diopter cylinder (DC) at 180 axes of the right eye and +3.00 DS and -0.5 DC at 180 axes of the left eye. Anterior segment and retinal examinations were unremarkable. In the primary position of gaze, the patient presented with 40 PD exotropia in the left eye, measured with the Krimsky prism test, and globe retraction of grade 1 of the left eye was observed (Figure 2B). There was no upshoot or downshoot of the left eye in the adduction position and he was orthotropic in the left gaze (Figure 2C). Adduction was severely limited in the left eye -4 while the extraocular motility was full in the right eye (Figure 2A). He was diagnosed with type II DRS in the left eye.

**Figure 3:**
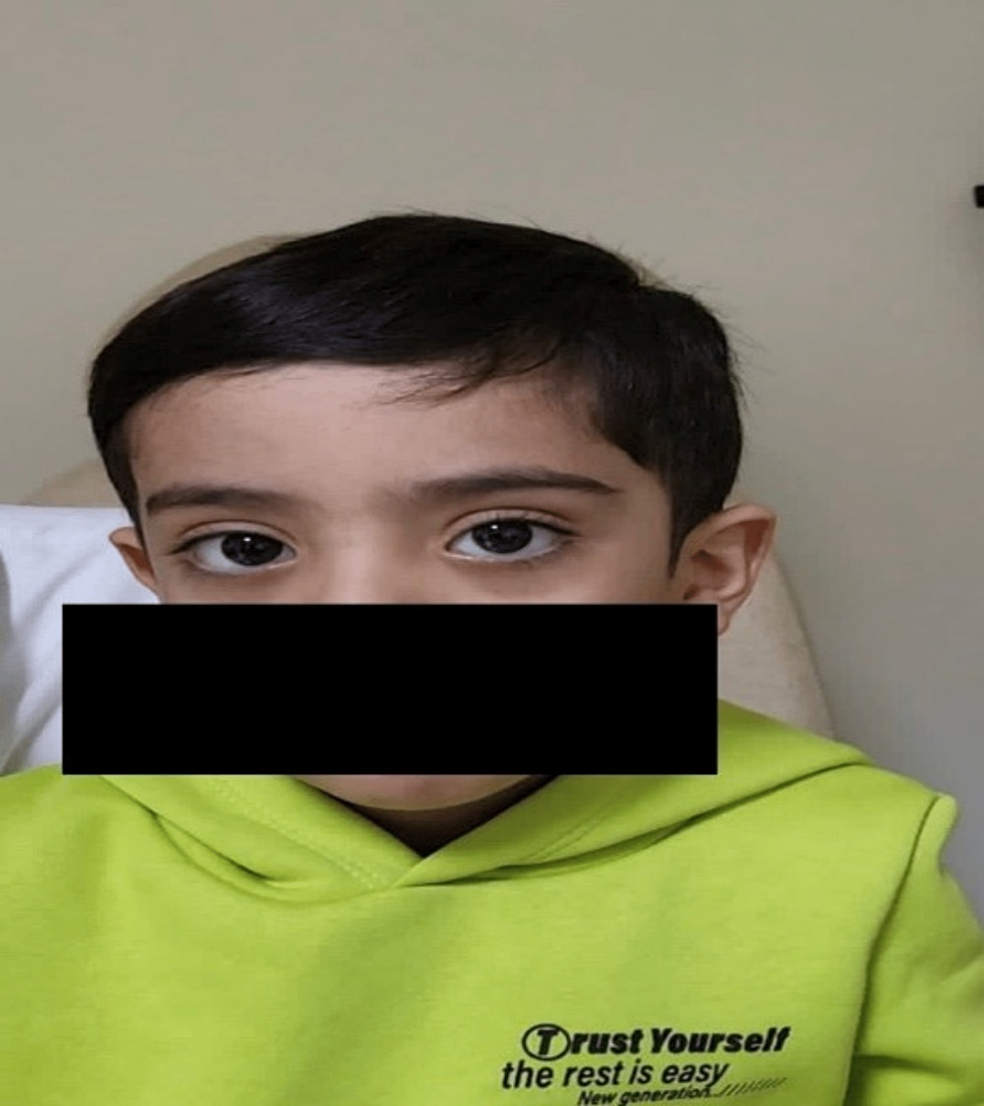
Postoperative evaluation showing improvement of abnormal head posture

**Figure 4:**
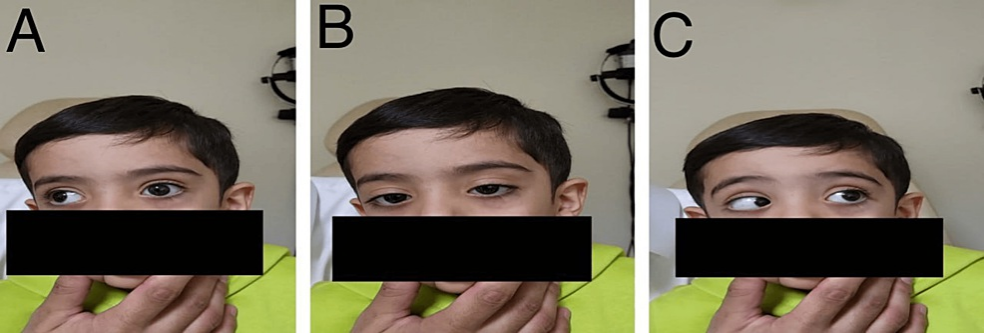
Postoperative assessment A. Improvement of adduction limitation B. Improvement of exotropia C. Mild limitation of abduction

A 7-mm lateral rectus recession was performed in both eyes. No extraocular muscle abnormalities were found. Postoperatively, the patient was orthotropic for distance and near in primary gaze with resolved face turn in three months (Figure 3). The limitation of adduction in the left eye postoperatively was improved to -2 (Figure 4A). However, some limitation of abduction -1 was observed (Figure 4C). The exotropia resolved in the primary position of gaze and remained in the right gaze (Figure 4B). Also, the globe retraction has improved in the adduction position of the left eye.

## Discussion

DRS type II is a rare congenital motility disorder affecting 7% of strabismic children [4]. Type II DRS is characterized by limited adduction, primary position exotropia, and normal or mild limited abduction [4]. It usually affects one eye, and the left eye is more likely to be affected than the right [5]. Our patient had complete limitation of adduction, as well as significant primary position exotropia and a significant large face turn. Abnormal head posture seems to be a compensatory mechanism for maintaining fusion. Akbari et al. found that all patients with exotropia had heads turned toward the opposite side of the affected eye [6]. In consistent, our case had a similar finding but with a large significant face turn. DRS is associated with systemic anomalies, which are 10 to 20 times more common in sporadic cases [4]. Our case did not have any systemic association, and it was sporadic. The prevalence of amblyopia in DRS has been estimated to be between 3% and 25%, with an average of 14% [7]. Our case had a strong preference toward the normal eye, indicating amblyopia of the Duane eye.

There have been a few case reports of type II DRS all over the world. Olawoye et al. reported a four-year-old girl who presented to the clinic with unilateral type II DRS of 12 PD in primary gaze. She has normal birth and development, and has a normal head posture; she was treated conservatively [8]. Our case was treated surgically due to a large right-sided face turn and 40 PD exotropia in the primary position. Mao et al. reported the nine-year-old boy was presented to the clinic with 15 PD exotropia in the primary position and a 40° right-sided face turn at a distance; he was diagnosed with type II DRS and planned for surgery in the unaffected eye [9]. Postoperatively, the patient was orthotropic in the primary position with a resolved face turn in six months [9]. Akbari et al. found the largest angle of deviation to be 35 PD, which was treated with bilateral lateral rectus recession [6]. Our case was diagnosed with type II DRS in the left eye and was treated with bilateral lateral rectus recession. The facial turn had resolved three months after surgery and was orthotropic for distance and near in the primary position, but he developed a -1 limitation of abduction in the Duane eye which is not routinely observed after a 7-mm lateral rectus recession.

The surgical management of type II DRS has been considered more challenging compared with other types of DRS. There are many options for the treatment of DRS type II, including unilateral or bilateral lateral rectus recession and vertical rectus transposition with possible medial rectus resection in resistant cases [3]. The surgical indication for type II DRS is poor head position, significant primary gaze deviation, extensive upshoot, and downshoot [3]. Because DRS type II is uncommon, it is important to recognize its primary symptoms and signs and to rule out other possible diagnoses, such as Inverse Duane's Retraction Syndrome.

## Conclusions

Type II DRS is a rare ocular motility disorder that accounts for 7% of all strabismus. As far as we know, this is the first isolated type II DRS reported in Saudi Arabia. DRS management is difficult, and the surgical approach to such a patient must be tailored to the degree of ocular deviation, abnormal head position, globe retraction, and overshoots. Lateral rectus muscle recession is expected to have a good outcome.

## References

[1] Kekunnaya R, Negalur M (2017). Duane retraction syndrome: causes, effects and management strategies. Clin Ophthalmol.

[2] DeRespinis PA, Caputo AR, Wagner RS, Guo S (1993). Duane's retraction syndrome. Surv Ophthalmol.

[3] Gaur N, Sharma P (2019). Management of Duane retraction syndrome: a simplified approach. Indian J Ophthalmol.

[4] Muni I, Kumar B (2022). Duane Retraction Syndrome. https://www.ncbi.nlm.nih.gov/books/NBK570558/.

[5] Mohan K, Sharma A, Pandav SS (2008). Differences in epidemiological and clinical characteristics between various types of Duane retraction syndrome in 331 patients. J AAPOS.

[6] Akbari MR, Masoumi A, Masoomian B, Mirmohammadsadeghi A, Mehrpour M (2020). Surgical outcome of patients with unilateral exotropic Duane retraction syndrome. J AAPOS.

[7] Yüksel D, Orban de Xivry JJ, Lefèvre P (2010). Review of the major findings about Duane retraction syndrome (DRS) leading to an updated form of classification. Vision Res.

[8] Olawoye OO, Olusanya BA, Baiyeroju AM (2014). Duane retraction syndrome in a Nigerian child. Pan Afr Med J.

[9] Mao K, Yan X, Ding K, Chen L, Lin X (2021). Contralateral lateral rectus muscle recession in a patient with unilateral exotropic Duane retraction syndrome type II: a case report. Strabismus.

